# Smoking Modifies the Associated Increased Risk of Future Cardiovascular Disease by Genetic Variation on Chromosome 9p21

**DOI:** 10.1371/journal.pone.0085893

**Published:** 2014-01-22

**Authors:** Viktor Hamrefors, Bo Hedblad, George Hindy, J. Gustav Smith, Peter Almgren, Gunnar Engström, Marketa Sjögren, Klas Gränsbo, Marju Orho-Melander, Olle Melander

**Affiliations:** 1 Department of Clinical Sciences, Faculty of Medicine, Lund University, Malmö, Sweden; 2 Clinical Physiology and Nuclear Medicine Unit, Skåne University Hospital, Malmö, Sweden; 3 Department of Cardiology, Faculty of Medicine, Lund University, Lund, Sweden; 4 Department of Cardiology, Skåne University Hospital, Lund, Sweden; 5 Program in Medical and Population Genetics, Broad Institute of Harvard and MIT, Cambridge, Massachusetts, United States of America; 6 Department of Internal Medicine, Skåne University Hospital, Malmö, Sweden; Medical University Hamburg, University Heart Center, Germany

## Abstract

**Aims:**

Genetic predisposition for cardiovascular disease (CVD) is likely to be modified by environmental exposures. We tested if the associated risk of CVD and CVD-mortality by the single nucleotide polymorphism rs4977574 on chromosome 9p21 is modified by life-style factors.

**Methods and results:**

A total of 24944 middle-aged subjects (62% females) from the population-based Malmö-Diet-and-Cancer-Cohort were genotyped. Smoking, education and physical activity-levels were recorded. Subjects were followed for 15 years for incidence of coronary artery disease (CAD; N = 2309), ischemic stroke (N = 1253) and CVD-mortality (N = 1156). Multiplicative interactions between rs4977574 and life-style factors on endpoints were tested in Cox-regression-models. We observed an interaction between rs4977574 and smoking on incident CAD (P = 0.035) and CVD-mortality (P = 0.012). The hazard ratios (HR) per risk allele of rs4977574 were highest in never smokers (N = 9642) for CAD (HR = 1.26; 95% CI 1.13–1.40; P<0.001) and for CVD-mortality (HR = 1.40; 95% CI 1.20–1.63; P<0.001), whereas the risk increase by rs4977574 was attenuated in current smokers (N = 7000) for both CAD (HR = 1.05; 95%CI 0.95–1.16; P = 0.326) and CVD-mortality (HR = 1.08; 95%CI 0.94–1.23; P = 0.270). A meta-analysis supported the finding that the associated increased risk of CAD by the risk-allele was attenuated in smokers. Neither education nor physical activity-levels modified the associated risk of CAD, ischemic stroke and CVD mortality conferred by rs4977574.

**Conclusion:**

Smoking may modify the associated risk of CAD and CVD-mortality conferred by genetic variation on chromosome 9p21. Whether the observed attenuation of the genetic risk reflects a pathophysiological mechanism or is a result of smoking being such a strong risk-factor that it may eliminate the associated genetic effect, requires further investigation.

## Introduction

Family history is a well recognized important risk factor for cardiovascular disease (CVD) [1]. Similar to most other common diseases, the inheritance of CVD is multifactorial, with genetic and environmental factors and interactions between them affecting the risk [2].

Genome wide association studies (GWAS) have been successful in identifying common genetic factors that associate with multifactorial diseases including CVD [3], [4]. Single nucleotide polymorphisms (SNPs) on chromosome 9p21 have been found to strongly associate with coronary artery disease (CAD) and myocardial infarction (MI) in the population, with risk allele frequencies of around 50% in populations of European ancestry and odds ratios for CAD and MI of ∼ 1.30 per allele [5]–[7]. The association of these SNPs with CAD and MI has been confirmed in numerous populations of European ancestry [4], [8], [9] and in other ethnicities [10]–[11]. Beyond CAD and MI, the same SNPs on Chromosome 9p21 associate with other CVD manifestations, including ischemic stroke [12]. Importantly, the chromosome 9p21 SNPs have been found not to associate with any of the traditional cardiovascular risk factors [5]–[7].

It is likely that the associated effect of genetic factors on CVD is modified by different environmental exposures [13]. Today, a number of modifiable environmental and life-style related risk factors show consistent evidence as risk factors for CVD. These include tobacco smoking, a low socioeconomic status, often measured as a low educational level, and physical inactivity [14]. Accounting for the complex nature of CVD, knowledge of how such life-style related risk factors may interact with genetic susceptibility variants on CVD risk is important for CVD risk prediction and prevention [15], [16]. However, very little is known about such putative gene-environment interactions.

In this study we tested whether the associated increased risk of future CVD and CVD-mortality by the common CVD risk SNP on chromosome 9p21 (rs4977574) is modified by life-style risk factors including smoking, educational level and physical activity level. We tested this hypothesis in 24944 middle aged Swedish subjects from the Malmö Diet and Cancer Cohort Study (MDCS), with around 15 years follow-up.

## Methods

### Study population

MDCS is a prospective population-based cohort study that initially recruited a total of 30447 subjects during the years 1991–1996. Subjects born between 1923 and 1950 living in the city of Malmö in Sweden were eligible for participation [17]. At baseline, participants underwent measurement of anthropometric variables and blood pressure, and provided blood samples. Subjects were also asked to complete a self-administered questionnaire of health and life-style related factors, including current and previous disease, medication, tobacco smoking, education and physical activity.

**Figure 1:**
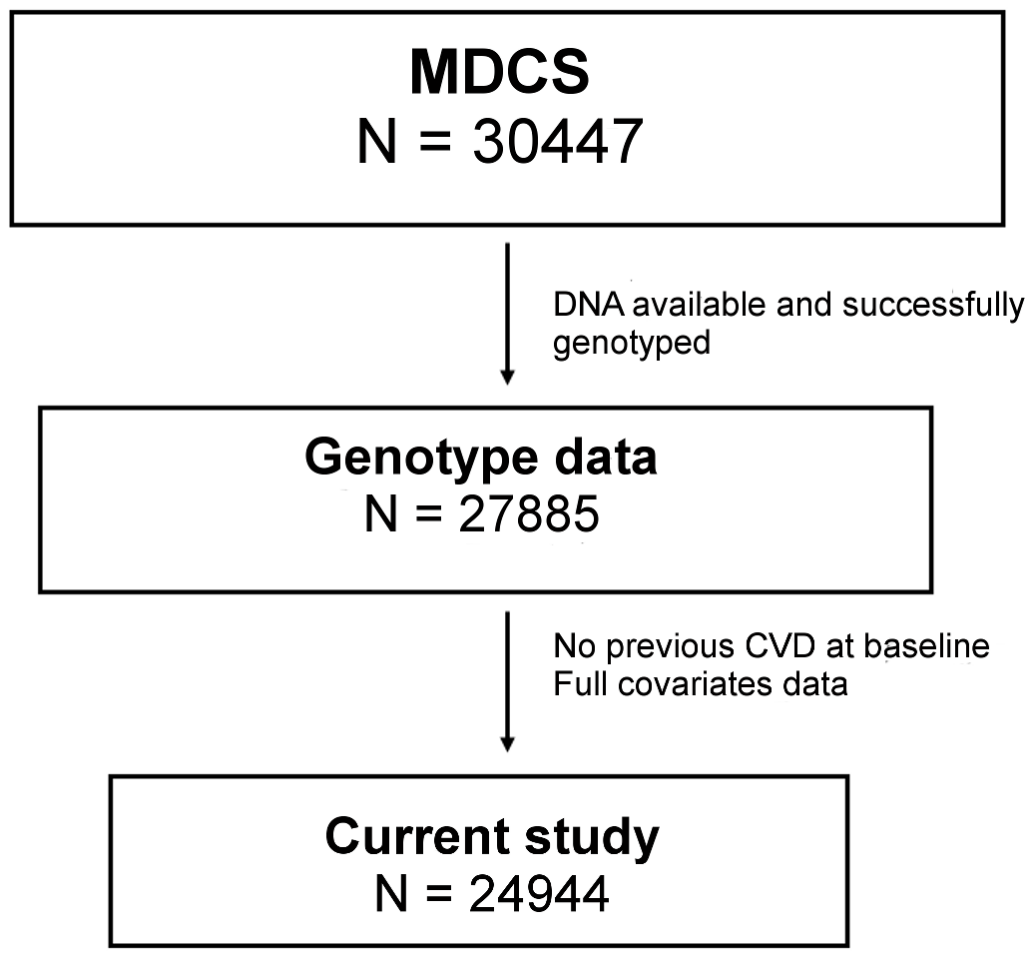
Selection of MDCS subjects. The current study included subjects with stored DNA who were successfully genotyped for rs4977574 on chromosome 9p21 and who had no previous history CVD at baseline and complete covariate data (age, sex, smoking status, education, physical activity, systolic blood pressure, antihypertensive medication and BMI). MDCS  =  Malmö Diet and Cancer Study.

DNA was extracted and successfully genotyped for the rs4977574 in 27885 subjects in MDCS. After excluding subjects with previous CVD at baseline (i.e. a history of MI, coronary-artery-by-pass graft surgery (CABG), percutaneous coronary intervention (PCI), or stroke) a total of 26855 subjects remained. Of these, we selected subjects that had complete baseline data for all variables and covariates of interest including smoking status, educational level, physical activity, systolic blood pressure, use of antihypertensive medication and body mass index (BMI), leaving us with a total of 24944 subjects for the current study (Figure 1).

The study was approved by the Regional Ethical Review Board in Lund at Lund University, Sweden. Written informed consent was obtained from all participants.

### Assessment of end-points

The three primary endpoints of our study were CAD, ischemic stroke and CVD-mortality (defined below). The endpoints were identified through linkage of the 10-digit personal identification number of each Swedish citizen with four registers: the Swedish Hospital Discharge Register, Swedish Coronary Angiography and Angioplasty Registry (SCAAR), the Stroke Register of Malmö and the Swedish Cause of Death Register. The registers have been previously described and validated for classification of outcomes [18]–[21]. Follow-up for the study extended to June 30, 2009.

CAD was defined as fatal or non-fatal MI, death from ischemic heart disease, CABG or PCI. MI was defined on the basis of International Classification of Diseases 9th and 10th Revisions (ICD9 and ICD10) codes 410 and I21, respectively. Death due to ischemic heart disease was defined on the basis of codes 412 and 414 (ICD9) or I22–I23 and I25 (ICD10). CABG was identified from national Swedish classification systems of surgical procedures, the KKÅ system from 1963 until 1989 and the Op6 system since then. CABG was defined as a procedure code of 3065, 3066, 3068, 3080, 3092, 3105, 3127, 3158 (Op6) or FN (KKÅ97). PCI was defined based on the operation codes FNG05 and FNG02. Fatal or nonfatal stroke was assessed using codes 430, 431, 434 and 436 (ICD9) and I60, I61, I63, and I64 (ICD10). Hemorrhagic strokes were however censored in the analyses, meaning that only cerebral infarctions (code 434 for ICD9/I63 for ICD 10) were included in the endpoint definition. CVD-mortality was defined as underlying cause of death classified as ICD-9 diagnoses 390–459 and ICD-10 diagnoses I00-199.

### Genotyping and definition of the independent variables at baseline

In MDCS, DNA was extracted from frozen granulocyte or buffy coat samples from blood from the baseline examination using QIAamp 96 spin blood kits (QIAGEN, VWR, Gaithersburg, MD, USA). The rs4977574 SNP (A/G) on chromosome 9p21 was genotyped using “Assay by design” TaqMan probes with a real time polymerase chain reaction assay on an ABI-7900HT equipment (Applied Biosystems, Foster City, CA) according to the manufacturer’s standard protocols. 20% of the samples were run in duplicate as part of the quality control process and the concordance was > 99.9%. The number of rs4977574 risk alleles (G) for each subject was coded as a linear variable assuming an additive effect.

The status of smoking was self-reported and coded as 0  =  never, 1 =  former or 2  =  current (i.e. any smoking within the past year) in a categorical variable. Passive smoking was defined as exposure to smoking either at home (“Do the persons you live with smoke indoors, or have they done so previously?”) or at work (“Do you regularly stay in places of work [apart from your home] where people smoke, or have you previously been staying in such places regularly?”) and was coded as a dichotomous variable. Education was defined as the self-reported highest level of education and coded as a six-graded categorical variable (0  =  did not complete elementary school, 1  =  elementary school (6–8 yrs), 2  =  junior secondary school (9–10 yrs), 3  =  education at advanced level (12 yrs); 4  =  at least one additional year, 5  =  university degree). For physical activity the information reported by the study participants for leisure-time physical activity level during the preceding year was used. A summary score of all physical activities was obtained by using intensity factors for each activity combined with information on the time spent on the activity. This physical activity (PA)-score has previously been described in detail and it has been validated with an accelerometer monitor in a random sample of 369 subjects in MDCS [22].

### Statistics

Main effects as well as interactions were all tested in multivariable proportional-hazards models using Cox regression analysis to test associations between the independent variables and time to the first event of each end-point. The proportional-hazards assumption was confirmed by visual inspection of survival curves.

Evidence of multiplicative interaction between the number of rs4977574 risk alleles and smoking, educational level and physical activity on the end-points was tested by constructing Cox regression models that included the respective multiplicative interaction terms (rs4977574 x smoking status; rs4977574 x educational level; rs4977574 x quintiles of physical activity score) in addition to the main effect terms. The likelihood ratio (LR) tests were performed comparing model fit with and without the interaction terms in order to test for evidence of significant interaction. We compared the fit of simple models adjusted for age and sex only, as well as models including additional covariates BMI, systolic blood pressure and use of antihypertensive medication in addition to all the three main effect terms. A P-value of less than 0.05 was considered significant.

For incident CAD, we also performed a meta-analysis including additional data from a recent report of interaction analyses between chromosome 9p21 variation and various environmental factors in 9877 subjects from the Atherosclerosis Risk in Communities (ARIC) Study [23]. The meta-analysis was performed on the study level, by pooling the effect estimates for the associated risk of incident CAD by the chromosome 9p21 risk locus in smokers and non-smokers respectively.

Statistical analyses were performed using SPSS Statistics 19.0–21.0 (SPSS Inc., Chicago, IL, USA) and Stata 11.0 (StataCorp LP, College Station, Texas, USA).

## Results

### Study population and incidence of CVD during follow-up

Characteristics of the study population according to genotype are shown in Table 1. For the incident end-points of CAD (n = 2309), ischemic stroke (n = 1253) and CVD-mortality (n = 1156) the subjects were followed for a median time of 14.5, 14.6 and 14.7 years, respectively.

**Table 1:** Population characteristics.

	Chromosome 9p21 rs4977574 genotype		
	*0 risk alleles A/A*	*1 risk allele A/G*	*2 risk alleles G/G*
**Total subjects, n (%)**	7609	12311	5024
Men	2881 (37.9)	4682 (38.0)	1892 (37.7)
Women	4728 (62.1)	7629 (62.0)	3132 (62.3)
**Age, years**	58.0 (7.7)	58.0 (7.6)	57.9 (7.7)
**Smoking status, n (%)**			
Never smokers	2896 (38.1)	4737 (38.5)	2010 (40.0)
Former smokers	2537 (33.3)	4105 (33.3)	1658 (33.0)
Current smokers	2176 (28.6)	3469 (28.2)	1356 (27.0)
**Highest level of education, n (%)**			
No elementary school	57 (0.7)	95 (0.8)	51 (1.0)
Elementary school (6–8 yrs)	3084 (40.5)	5024 (40.8)	2015 (40.1)
Junior Sec. School (9–10 yrs)	2015 (26.5)	3283 (26.7)	1260 (25.1)
Advanced level (12 yrs)	656 (8.6)	1084 (8.8)	497 (9.9)
At least one additional year	671 (8.8)	1086 (8.8)	457 (9.1)
University degree	1126 (14.8)	1739 (14.1)	744 (14.8)
**Low physical activity, n (%)** *	1492 (19.6)	2355 (19.1)	1011 (20.1)
**Systolic blood pressure, mmHg**	141.0 (20.0)	141.0 (20.0)	141.4 (20.2)
**Use of AHT, n (%)**	1321 (17.4)	2023 (16.4)	821 (16.3)
**BMI, kg/m^2^**	25.8 (4.0)	25.7 (4.0)	25.7 (4.0)
**Incidence of events during follow-up**			
CAD, events (events/1000 p-ys)	633 (6.0)	1134 (6.7)	542 (7.9)
Ischemic Stroke, events (events/1000 p-ys)	355 (3.3)	609 (3.5)	289 (4.1)
Cardiovascular mortality, events (events/1000 p-ys)	310 (2.8)	586 (3.4)	260 (3.6)

Mean (SD) if not stated other.

p-ys =  person-years.

* Defined as the lowest quintile of the Physical Activity score in MDCS.

Subjects in MDCS excluded from the current study (N = 5503) because of incomplete genotype data, covariate data and/or previous CVD at baseline (Figure 1) generally had a higher burden of cardiovascular risk factors (more likely to be males, less likely to be never-smokers, slightly higher systolic blood pressure and BMI) compared to included subjects. In accordance with these observations, there was also a higher incidence of end points in excluded subjects (Table S1A in File S1). Most of the higher CVD risk burden in excluded subjects could be attributed to subjects with previous CVD at baseline. Compared to included subjects and to excluded subjects without previous CVD, these subjects were much more likely to be men and former smokers; they were older, had higher blood pressure despite an extensive use of antihypertensive medication and they had higher BMI. As expected, the incidence of CAD, ischemic stroke and CVD death was considerably higher in subjects with previous CVD. (Table S1B in File S1).

### Main effects of the independent variables on CVD incidence

In Cox regression models adjusted for age and sex the rs4977574 associated with all three end-points with hazard ratios of 1.12–1.16 per risk allele. Current smoking showed a strong association with all three end-points, whereas former smoking was associated with incident CAD and CVD-mortality, but not with incident ischemic stroke. Level of education was associated with incident ischemic stroke, and a significant trend for association also with incident CAD and CVD-mortality could be observed across education categories. Level of physical activity showed non-linear associations with all end-points (Table 2). Results for main effects were similar in the multivariate adjusted models (Table S2 in File S1).

**Table 2:** Main effects.

	CAD HR (95% CI)	Ischemic Stroke HR (95% CI)	Cardiovascular mortality HR (95% CI)
**rs4977574, per allele**	1.16 (1.09–1.23)	1.12 (1.04–1.22)	1.14 (1.05–1.24)
Smoking status*			
Former smoker	1.21 (1.09–1.34)	1.02 (0.89–1.17)	1.27 (1.09–1.48)
Current smoker	2.01 (1.8–2.23)	1.65 (1.44–1.89)	2.67 (2.31–3.09)
Highest education*			
Elementary school (6–8 yrs)	0.97 (0.63–1.50)	0.53 (0.34–0.82)	0.82 (0.46–1.45)
Junior Sec. School (9–10 yrs)	0.81 (0.52–1.25)	0.45 (0.29–0.71)	0.68 (0.38–1.22)
Advanced level (12 yrs)	0.75 (0.48–1.17)	0.38 (0.23–0.62)	0.58 (0.32–1.07)
At least one additional year	0.75 (0.48–1.18)	0.40 (0.24–0.64)	0.54 (0.29–0.99)
University degree	0.63 (0.40–0.98)	0.35 (0.22–0.57)	0.55 (0.30–1.00)
*P for trend*	*<0.001*	*<0.001*	*<0.001*
Quintiles of PA score*			
Q2	0.68 (0.60–0.78)	0.70 (0.59–0.83)	0.62 (0.52–0.75)
Q3	0.70 (0.62–0.80)	0.70 (0.59–0.83)	0.64 (0.54–0.77)
Q4	0.73 (0.64–0.83)	0.63 (0.53–0.75)	0.63 (0.53–0.75)
Q5	0.75 (0.67–0.85)	0.66 (0.56–0.78)	0.61 (0.51–0.72)

Adjusted for age and sex.

PA score  =  Physical Activity Score.

* Hazard ratio (HR) in relation to the first category in the categorical variables (never smokers, “did not complete elementary school”, and Q1 of PA-score respectively).

### Interaction between Chromosome 9p21 risk alleles and smoking status

We observed a significant interaction between the number of rs4977574 risk alleles and smoking status on incidence of CAD (P_interaction_  =  0.035) and CVD-mortality (P_interaction_  =  0.012). These interactions remained significant in the fully adjusted models for both incident CAD (P_interaction_  =  0.035) and CVD-mortality (P_interaction_  =  0.029). No interaction was observed between the rs4977574 risk allele and smoking status on incident ischemic stroke (P_interaction_  =  0.702).

**Figure 2:**
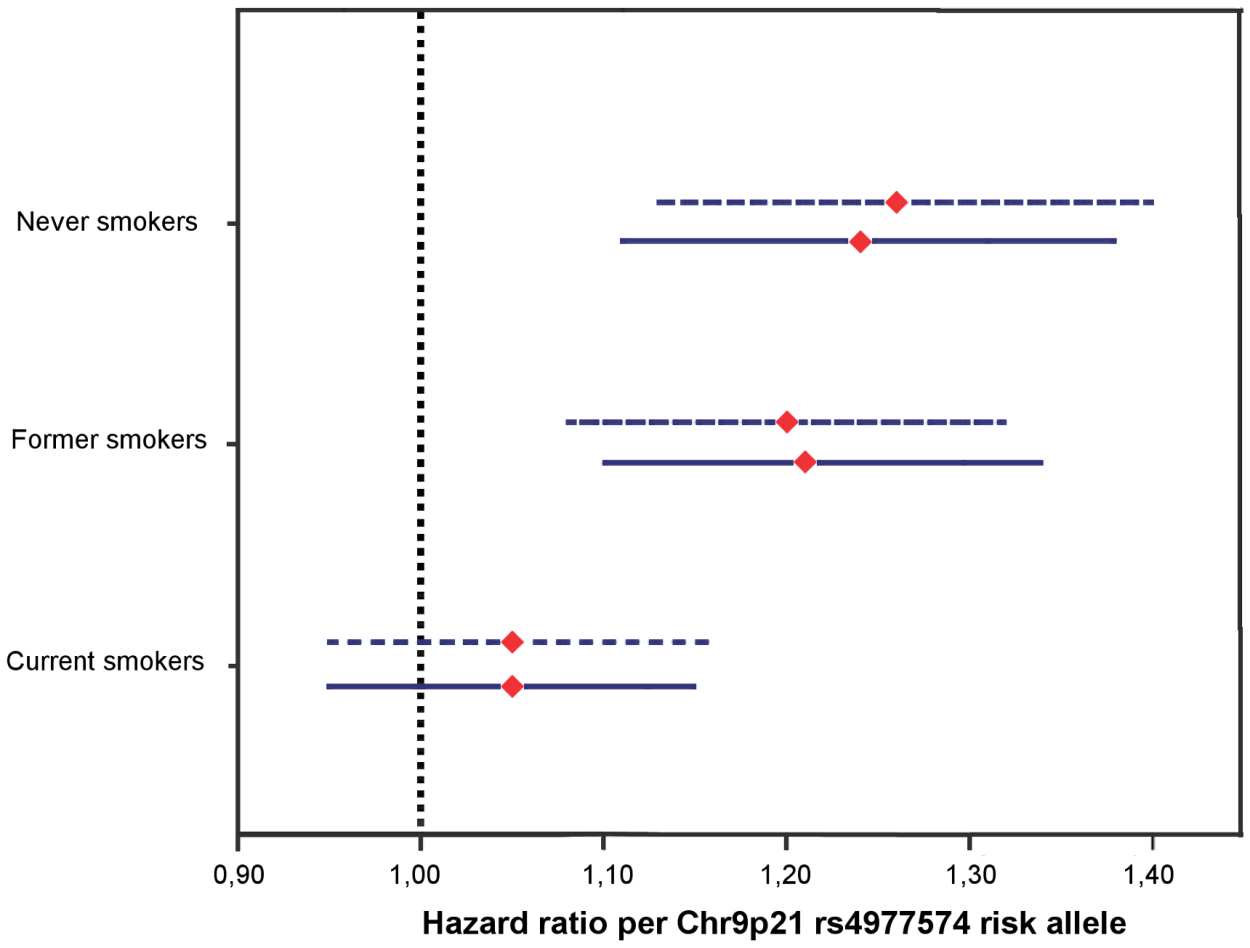
Risk of incident CAD by rs4977574 stratified by smoking status. Hazard ratios (HR) with 95% Confidence Interval per risk allele of rs4977574 in never (N = 9642) former (N  = 8300) and current (N = 7000) smokers respectively. Models adjusted for age and sex (dotted upper lines) and adjusted for covariates age, sex, smoking status, education, physical activity, systolic blood pressure, antihypertensive medication and BMI (continuous lower lines).

**Figure 3:**
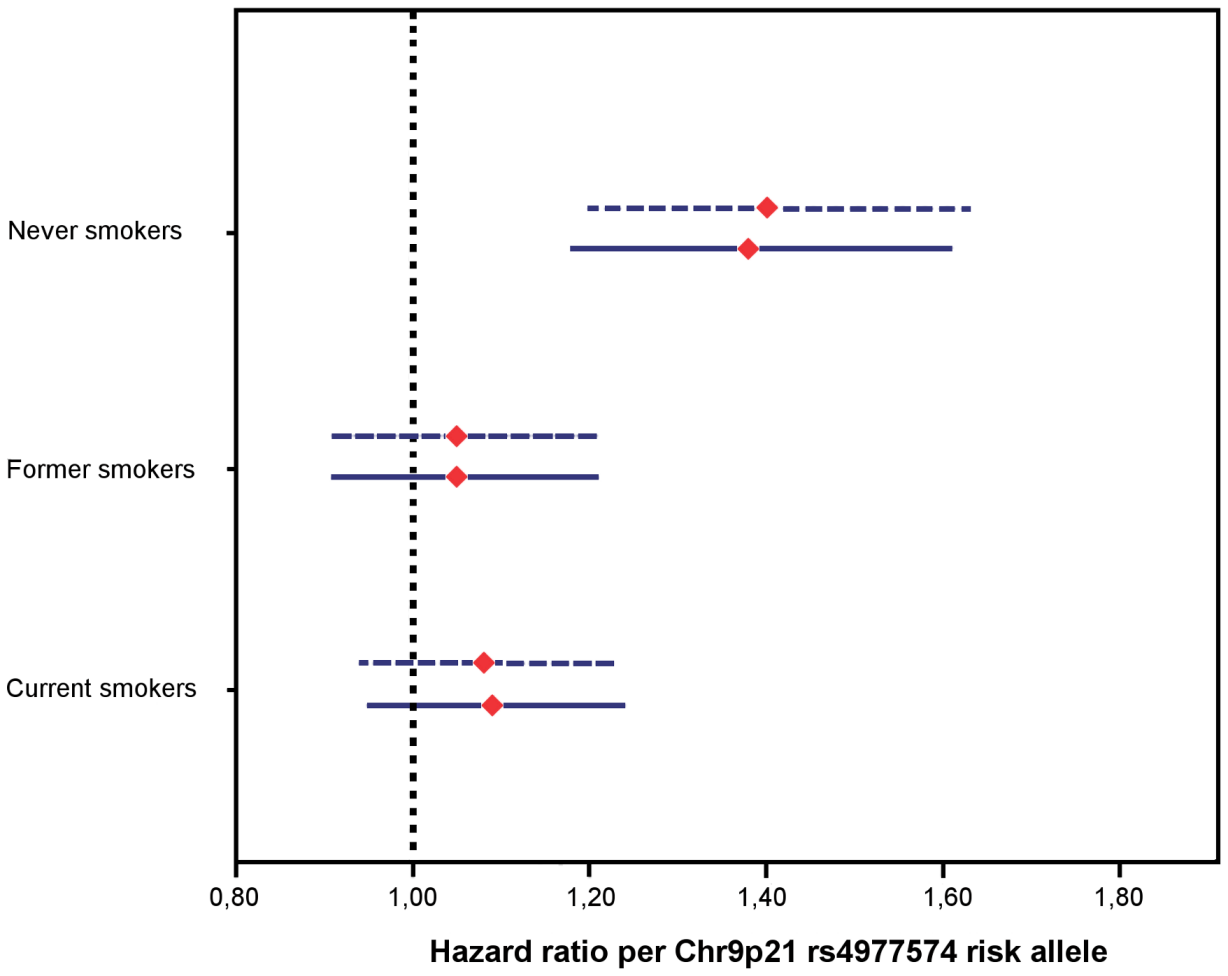
CVD-mortality by rs4977574 stratified by smoking status. Hazard ratios (HR) with 95% Confidence Interval per risk allele of rs4977574 in never (N = 9642) former (N = 8297) and current (N = 7000) smokers respectively. Models adjusted for age and sex (dotted upper lines) and adjusted for covariates age, sex, smoking status, education, physical activity, systolic blood pressure, antihypertensive medication and BMI (continuous lower lines).

As we found significant interactions between rs4977574 and smoking status on incident CAD and CVD-mortality, we tested the associated effect of rs4977574 on these two endpoints according to smoking status (Figures 2–3). For incident CAD, the associated effect of rs4977574 was found to be highly significant in never-smokers (HR 1.26 per risk allele; 95% CI 1.13–1.40; P<0.001) and former smokers (HR = 1.20 per risk allele; 95% CI 1.08–1.32; P<0.001), whereas this associated effect was attenuated and not significant in current smokers (HR = 1.05 per risk allele; 95% CI 0.95–1.16; P = 0.326) (Table 3, Figure 2). Since we had additional data also on the exposure to passive smoking for 22049 subjects we performed stratification within the groups of never and former smokers according to this variable. In never smokers, the significant associated effect of rs4977574 risk alleles on incident CAD was attenuated among subjects that reported passive exposure to smoking (HR 1.14 per risk allele; 95% CI 0.99–1.32; P = 0.068), contrasting to subjects that were not exposed to passive smoking, in whom rs4977574 showed a high hazard ratio per allele (HR 1.56 per risk allele; 95% CI 1.29–1.88; P<0.001). The results were similar in the adjusted models and were similar in both sexes (Tables S3A-B in File S1). In current smokers we had information also on baseline “pack-years” (number of cigarette packs per day x years of smoking; N = 6256) and cigarettes smoked per day (N = 6311). Thus, within the group of current smokers we additionally stratified for pack-years and number of daily cigarettes in order to test if there was a suggestive dose-relationship for the modification of the genetic effect by smoking. There was however no such evident pattern for pack-years or number of daily cigarettes further modifying the chromosome 9p21 genetic association for incident CAD. (Table 3)

**Table 3:** Risk of incident CAD by rs4977574 stratified by smoking status.

	Events (total cases)	rs4977574 HR per allele	95% CI	P-value
**Never smokers**	**675 (9642)**	**1.26**	**1.13**–**1.40**	**<0.001**
No passive smoking	220 (3339)	1.56	1.29–1.88	<0.001
Passive smoking	379 (5069)	1.14	0.99–1.32	0.068
**Former smokers**	**814 (8300)**	**1.20**	**1.08**–**1.32**	**<0.001**
No passive smoking	177 (2146)	1.30	1.05–1.60	0.015
Passive Smoking	528 (5221)	1.19	1.06–1.35	0.004
**Current smokers**	**820 (7000)**	**1.05**	**0.95**–**1.16**	**0.326**
Pack-years < median	298 (3090)	1.05	0.89–1.23	0.572
Pack-years ≥ median	407 (3165)	1.02	0.89–1.18	0.751
Daily Cigs < median	321 (3057)	0.97	0.83–1.13	0.704
Daily Cigs ≥ median	390 (3253)	1.08	0.93–1.24	0.317

Adjusted for age and sex.

Daily cigs  =  number of daily cigarettes.

For CVD-mortality (Figure 3), the associated effect of rs4977574 was found to be highly significant only in the group of never smokers (HR 1.40 per risk allele; 95% CI 1.20–1.63;P<0.001), whereas the associated effect was attenuated and not significant among both current (HR 1.08 per risk allele; 95% CI 0.94–1.23; P = 0.270) and former smokers (HR 1.05 per risk allele; 95% CI 0.91–1.21; P = 0.525) (Table 4, Figure 3). The highest HRs were observed in never smokers that were not exposed to passive smoking. Including covariates in the models did not change the results which were similar in both sexes (Tables S4A-B in File S1). In contrast to the results for CAD, there was a suggestive pattern for a dose-response association modifying the genetic effect within current smokers, as the genetic effect seemed to be attenuated to a larger extent in subjects with more extensive smoking habits. (Table 4).

**Table 4:** CVD-mortality by rs4977574 stratified by smoking status.

	Events (total cases)	rs4977574 HR per allele	95% CI	P-value
**Never smokers**	**327 (9642)**	**1.40**	**1.20**–**1.63**	**<0.001**
No passive smoking	113 (3339)	1.78	1.37–2.32	<0.001
Passive smoking	174 (5070)	1.27	1.03–1.57	0.025
**Former smokers**	**383 (8297)**	**1.05**	**0.91**–**1.21**	**0.525**
No passive smoking	88 (2145)	1.38	1.02–1.85	0.034
Passive smoking	247 (5218)	0.96	0.81–1.15	0.676
**Current smokers**	**446 (7000)**	**1.08**	**0.94**–**1.23**	**0.270**
Pack-years < median	154 (3089)	1.20	0.96–1.50	0.11
Pack-years ≥ median	242 (3165)	1.00	0.84–1.21	0.972
Daily cigs < median	179 (3057)	1.14	0.93–1.41	0.214
Daily cigs ≥ median	218 (3252)	1.03	0.85–1.25	0.775

Adjusted for age and sex.

Daily cigs  =  number of daily cigarettes.

The fact that the associated risk of CAD and CVD-mortality by rs4977574 was attenuated in current smokers provided the rationale for studying also if the risk of incident events associated with smoking would be less in risk allele carriers. As expected, smoking was observed to be a strong risk factor regardless of genotype. However, we did observe a pattern of smoking having a less effect on risk of incident CAD and CVD-mortality in rs4977574 risk allele carriers compared to non-risk allele carriers (Tables 5–6).

**Table 5:** Smoking as a risk factor for incident CAD, stratified by number of rs4977574 risk alleles.

rs4977574 risk alleles	Events (total cases)	Former smoker HR (95% CI)	P	Current smoker HR (95% CI)	P
0	633 (7608)	1.24 (1.01–1.52)	0.038	2.21 (1.81–2.70)	<0.001
1	1134 (12309)	1.24 (1.07–1.45)	0.005	2.21 (1.90–2.56)	<0.001
2	542 (5024)	1.12 (0.92–1.38)	0.265	1.48 (1.19–1.84)	<0.001

Adjusted for age and sex.

**Table 6:** Smoking as a risk factor for CVD-mortality, stratified by number of rs4977574 risk alleles.

rs4977574 risk alleles	Events (total cases)	Former smoker HR (95% CI)	P	Current smoker HR (95% CI)	P
0	310 (7604)	1.76 (1.30–2.38)	<0.001	3.35 (2.48–4.52)	<0.001
1	586 (12309)	1.20 (0.96–1.48)	0.106	2.77 (2.26–3.39)	<0.001
2	260 (5024)	1.04 (0.77–1.42)	0.781	2.00 (1.48–2.70)	<0.001

Adjusted for age and sex.

### Meta-analysis: Risk of incident CAD by Chromosome 9p21 stratified by smoking status

**Figure 4:**
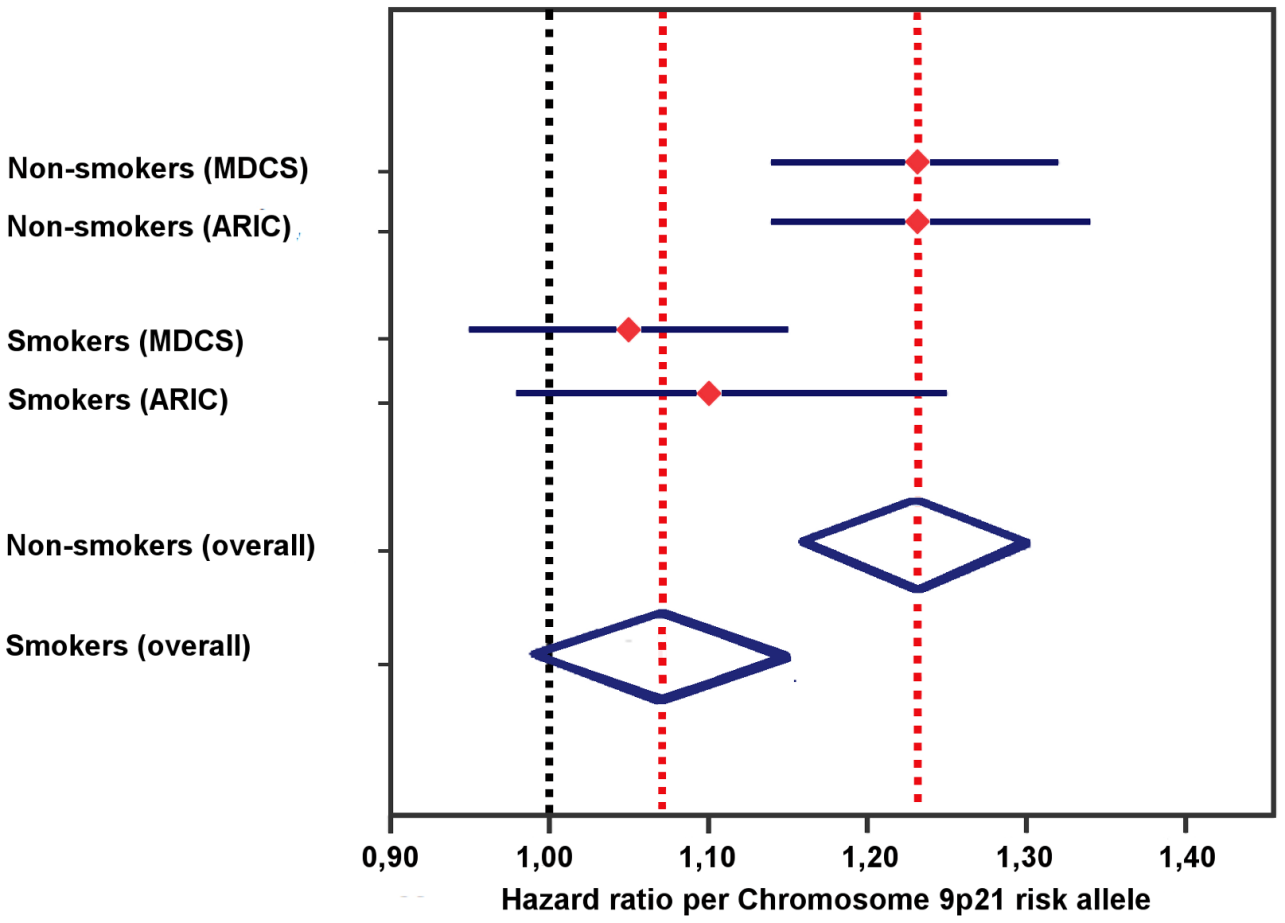
Meta analysis: Risk of incident CAD by Chromosome 9p21 stratified by smoking status. Hazard ratios (HR) with 95% Confidence Interval (CI) per Chromosome 9p21 risk allele in MDCS (rs4977574; N = 24944; 28% smokers) and ARIC (rs10757274; N = 9877; 25% smokers). Pooled HR per allele for incident CAD in non-smokers and smokers were 1.23 (95% CI 1.16–1.30) and 1.07 (95% CI 0.99–1.15) respectively. MDCS  =  Malmö diet and cancer study. ARIC  =  Atherosclerosis Risk in Communities Study.

In the meta-analysis of MDCS and ARIC, the associated risk of incident CAD by the chromosome 9p21 locus was found to be attenuated in smokers (Overall HR per allele  =  1.07; 95% CI 0.99–1.15). Contrary, when pooling the results from MDCS and ARIC in non-smokers (i.e. never and former smokers) there was an increased risk of incident CAD by chromosome 9p21 (Overall HR per allele  =  1.23; 95% CI 1.16–1.30) (Figure 4).

### Interaction between Chromosome 9p21 risk alleles, education and physical activity

No interactions were observed between rs4977574 and educational level or physical activity on incident CAD (P = 0.082 and P = 0.457, respectively), incident ischemic Stroke (P = 0.876 and P = 0.251, respectively) or CVD-mortality (P = 0.681 and P = 0.286, respectively). Results were similar also after adjusting the models for putative confounders (Table S5 in File S1).

## Discussion

In this population-based prospective study we evaluated gene-environment interactions for one of the strongest reported cardiovascular risk SNPs rs4977574 on chromosome 9p21. We report a significant interaction with smoking for incidence of CAD and CVD-mortality, with similar results in both men and women.

During the approximately 15 years of follow-up time we observed a strong association between rs4977574 and risk of incident CAD in non-smokers, whereas the significance of rs4977574 was fully attenuated in current smokers (Figure 2). These findings were similar in both sexes (Tables S3A-B in file S1). Interestingly, when stratifying additionally for passive smoking within the group of non-smokers we could observe that rs4977574 had the highest hazard ratio for incident CAD in never smokers that were never exposed to passive smoking (Table 3). There was however no evidence for a dose-response relationship for the genetic effect modification by smoking within the group of current smokers (Table 3). The finding of an attenuation of the associated increased risk of CAD by the risk locus on chromosome 9p21 in smokers was further supported by a meta-analysis including recent results from ARIC [23] (Figure 4).

For CVD-mortality the results were similar to the result for CAD, showing that in relative terms the risk influence of rs4977574 was consistently highest in never-smokers who had no previous exposure to passive smoking at baseline. However, for CVD-mortality, attenuation of the genetic effect was observed also in former smokers. Furthermore there was a suggestive pattern of a dose-response genetic effect modification, as the genetic effect for chromosome 9p21 seemed to be more markedly attenuated in subjects who smoked more. (Table 4). Naturally, a similar dose-response-test would have been very interesting to perform within the group of former smokers, however unfortunately, baseline pack-year-data and daily cigarettes consumption was available only in current smokers.

The interactions between smoking and the chromosome 9p21 CVD risk locus on CAD and CVD-mortality were evident also from a reverse point of view. That is, although smoking was a strong risk factor regardless of genotype, we observed a pattern of the risk conferred by smoking being lower in the risk allele carriers (Tables 5–6), with similar results in both sexes (Tables S6–S7 in File S1).

The reported effect size for the chromosome 9p21 risk locus on CAD and MI is larger for early-onset than for later onset disease [5]. A question that arose from the current study results for CAD was thus if the observed modification of the genetic effect on CAD by smoking could be further influenced by age. In order to address this question we stratified the group of non-smokers / smokers according to age at baseline. These analyses did reveal a pattern of smoking seeming to be a more evident modifier of the genetic effect of rs4977574 in older subjects, compared to younger subjects (Table 7). A possible explanation is that the chromosome 9p21 CVD risk locus might confer a substantial relative risk for CAD in the low-risk group of younger subjects even in the presence of a concurrent strong risk factor in the form of smoking. That is – younger subjects are at such comparably low risk for CAD that the weaker genetic effects would be preserved even if they smoke. Contrary, in older subjects who by means of their age (and the risk factors that come with age) are already at much higher risk for CAD, the addition of yet another strong risk such as smoking may diminish the relative influence of genetic factors on risk of CAD to larger extent.

**Table 7:** Risk of incident CAD by rs4977574 stratified by smoking status and age.

	Events (total cases)	rs4977574 HR per allele	95% CI	P-value
**Non-smokers**	**1489 (17942)**	**1.22**	**1.14**–**1.32**	**<0.001**
Age < median	346 (8316)	1.23	1.06–1.43	0.006
Age > median	1143 (9624)	1.21	1.11–1.31	<0.001
**Smokers**	**820 (7000)**	**1.05**	**0.95**–**1.16**	**0.326**
Age < median	316 (4154)	1.15	0.99–1.34	0.070
Age > median	504 (2846)	0.98	0.86–1.11	0.693

Adjusted for (age) and sex.

Even though the meta analysis including the results from ARIC further supports the finding that the increased risk of incident CAD by the chromsome 9p21 CVD risk locus was attenuated in smokers, the fact that the ARIC-based study in itself did not reveal a significant interaction between chromosome 9p21 and smoking (P_interaction_  =  0.14) is worth discussing. The ARIC-study examined the SNP rs10757274 which is in strong LD with rs4977574 (r^2^ = 0.94 in data from 1000 genomes pilot 1; value obtained from SNP Annotation and proxy search (SNAP) from Broad Institute; http://www.broadinstitute.org/mpg/snap/) and the endpoints in both studies were similar. We suggest two possible main explanations for the difference of the results. First, the ARIC-study included less subjects (approximately 10 000 versus 25 000 subjects) and even though the mean follow-up time in the ARIC-study was longer than in our study (17 vs 14 years) there were less CHD/CAD events in the ARIC-study (1653 versus 2309 events). The absence of significance for the interaction term in the ARIC-study (P = 0.14) might thus well be a matter of power. Second, subjects from the ARIC-study were generally younger at baseline than subjects from MDCS included in our study. Based on the above reasoning of smoking being a more evident modifier of the genetic effect by chromosome 9p21 in older subjects, this may be an additional possible explanation for the lack of significant interaction in the ARIC-study.

The causes behind the observed interaction between chromosome 9p21 and smoking on risk of CVD from our results could be looked at from both a pathophysiological and genetic-epidemiological point of view. From a pathophysiological view the chromosome 9p21 CVD risk locus has been consistently found not to associate with conventional cardiovascular risk factors. Molecular studies have found that the locus involves a specific non-coding RNA, termed antisense non-coding RNA in the INK4 locus (ANRIL), which has been suggested to be an epigenetic regulator of other genes potentially involved in CVD pathophysiology [24]. To the best of our knowledge, there are no reported associations between ANRIL and smoking or the known pathophysiological pathways of smoking. However, considering that smoking is likely to affect the risk of CVD via multiple pathways, one could speculate that smoking and the chromosome 9p21 risk locus (and ANRIL) might act via at least partially same (yet unknown) pathway(s) on CVD, and that smoking in itself would be sufficient cause for disease.

The results could also be looked from a more genetic-epidemiological view. As outlined in the previous discussion of the influence of age on genetic effect modification, smoking is in itself a very strong risk factor for CVD - and smokers constitute a high risk group for all cardiovascular events. It is thus tempting to speculate that the relative influence of genetic factors on CVD risk could be attenuated in such a high risk group. Among individuals with low conventional risk of CVD, the relative effect of genetic factors might instead be accentuated. This argument is supported by the higher effect estimates of the chromosome 9p21 CVD risk locus for early onset cases of MI [5], in which conventional cardiovascular risk factors are usually less prominent than in later onset-cases of MI and CAD. Also, our age-stratification analysis revealing a suggestive pattern of a more evident genetic effect attenuation by smoking in older (higher risk) subjects compared to younger ( lower risk) subjects support this hypothesis, as already discussed.

Practically, our results and the reasoning above may indicate that genetic screening for CVD could in fact be valuable in younger subjects and in other low-risk groups where conventional cardiovascular risk factors are not as prominent. This hypothesis is supported by studies showing no or little value over conventional risk factors for the chromosome 9p21 and other CVD risk SNPs in predicting CVD in the general population [25], [26], whereas modestly improved CVD risk prediction has been reported in low to intermediate risk groups [25], [27]. Further prospective studies investigating the value of chromosome 9p21 risk alleles as predictors of CVD risk in groups with few, or successfully treated conventional risk factors, are warranted.

We are aware of that our study has a number of limitations that deserve to be mentioned. First, the MDCS cohort consists of subjects mainly of European ancestry, thus limiting the generalization of the results to populations of non-European ancestry. Also, community based cohorts involving self reported data might suffer from an uncertainty concerning the validity of the data, although this problem should be less in prospective studies than in case-control studies. Furthermore, we were not able to evaluate how a change of exposure to the environmental risk factors during the follow-up period may have affected the results, for example how the risk conferred by rs4977574 was changed in subjects that quitted smoking during the follow-up. Finally, from a statistical point of view, if strict Bonferroni-correction is applied in order to correct for multiple interaction tests, the significance of the interaction between rs4977574 and smoking on incident CAD (P = 0.035) would be attenuated. However, we do think that the conclusive results in the subsequent stratification as well as the results from the meta-analysis still provide adequate evidence for considering an interaction between and chromosome 9p21 and smoking on risk of CAD to be likely.

## Conclusion

In this large prospective gene-environment interaction study we observed that smoking attenuated the associated increased risk of incident CAD and CVD-mortality by rs4977574 on chromosome 9p21. A meta-analysis of 35 000 subjects further supported the finding that the associated increased risk of CAD by the risk locus on chromosome 9p21 was attenuated in smokers. Whether a specific pathophysiological mechanism can explain these findings remains to be explored. The results raise hypotheses regarding strategies for genetic cardiovascular risk prediction in the population, suggesting that genetic factors may have a relatively larger influence on CVD risk in conventionally assessed low-risk groups compared to high risk groups.

## Supporting Information

File S1 **Additional and extended tables with supplementary data.**
(DOC)
