# Supplementary material for: Smoking Modifies the Associated Increased Risk of Future Cardiovascular Disease by Genetic Variation on Chromosome 9p21
Source: PLoS One. 2014 Jan 22;9(1):e85893. doi: 10.1371/journal.pone.0085893 (PMC3899088; doi:10.1371/journal.pone.0085893)
Supplement: File S1 — Additional and extended tables with supplementary data. (DOC) [file pone.0085893.s001.doc]

SUPPORTING INFORMATION

**Table S1A**: Population characteristics

|  | Current study population  (N = 24944) | Subjects in MDCS excluded from current study  (N = 5503) ^a^ |
| --- | --- | --- |
| **Sex, n (%)** Men Women | 9455 (37.9)  15489 (62.1) | 2666 (48.4)  2837 (51.6) |
| **Age, years** | 58.0 (7.7) | 58.3 (7.2) |
| **Chromosome 9p21, n (%)**  0 risk alleles: A/A 1 risk allele: A/G 2 risk alleles: G/G | 7609 (30.5)  12311 (49.4)  5024 (20.1) | 911 (31.0)  1373 (46.7)  657 (22.3) |
| **Smoking status, n (%)**  Never smokers  Former smokers  Current smokers | 9643 (38.7)  8300 (33.3)  7001 (28.1) | 1176 (32.5)  1358 (37.5) 1086 (30.0) |
| **Highest education, n (%)** No elementary school Elementary school (6-8 yrs)  Junior Sec. School (9-10 yrs)  Advanced level (12 yrs)  At least one additional year  University degree | 203 (0.8)  10123 (40.6)  6558 (26.3)  2237 (9.0)  2214 (8.9)  3609 (14.5) | 40 (1.1)  1605 (45.1)  867 (24.4)  303 (8.5)  275 (7.7)  465 (13.1) |
| **Low physical activity, n (%)**^b^ | 4858 (19.5) | 765 (22.4) |
| **Systolic blood pressure, mmHg** | 141.0 (20.0) | 141.5 (20.1) |
| **Use of AHT, n (%)** | 4165 (16.7) | 1114 (20.2) |
| **BMI, kg/m^2^** | 25.7 (4.0) | 26.3 (4.3) |
| **Incidence of events during follow up** |  |  |
| CAD, total events (events/1000 p-ys) | 2309 (6.7) | 936 (13.2) |
| Ischemic Stroke, total events (events/1000 p-ys) | 1253 (3.6) | 440 (6.1) |
| Cardiovascular mortality, events (events/1000 p-ys) | 1156 (3.2) | 590 (7.8) |

^a^ Subjects from MDCS excluded from the current study because of missing genetic and / or covariates data. Proportion numbers are based on available data in excluded subjects.

^b^ Defined as the lowest quintile of the Physical Activity score in MDCS (for definition of Physical Activity Score,

(see the methods section)

**Table S1B: Population characteristics**

|  | Current study  population  (N = 24944) | Subjects in MDCS  excluded from the  current study  (N = 5503) ^a^ | |
| --- | --- | --- | --- |
|  |  | No CVD  at baseline  (N = 4381) | CVD  at baseline  (N = 1121) ^b^ |
| **Sex, n (%)** Men Women | 9455 (37.9)  15489 (62.1) | 1854 (42.3)  2527 (57.7) | 811 (72.3)  310 (27.7) |
| **Age, years** | 58.0 (7.7) | 57.2 (6.9) | 62.9 (6.6) |
| **Chromosome 9p21, n (%)**  0 risk alleles: A/A 1 risk allele: A/G 2 risk alleles: G/G | 7609 (30.5)  12311 (49.4)  5024 (20.1) | 613 (32.1)  893 (46.7)  405 (21.2) | 298 (29.0)  479 (46.6)  252 (24.5) |
| **Smoking status, n (%)**  Never smokers  Former smokers  Current smokers | 9643 (38.7)  8300 (33.3)  7001 (28.1) | 961 (37.0)  814 (31.4) 819 (31.6) | 215 (21.0)  544 (53.0)  267 (26.0) |
| **Highest education, n (%)** No elementary school Elementary school (6-8 yrs)  Junior Sec. School (9-10 yrs)  Advanced level (12 yrs)  At least one additional year  University degree | 203 (0.8)  10123 (40.6)  6558 (26.3)  2237 (9.0)  2214 (8.9)  3609 (14.5) | 27 (1.1)  1039 (41.0)  671 (26.4)  222 (8.8)  206 (8.1)  372 (14.7) | 13 (1.3)  566 (55.6)  196 (19.3)  81 (8.0)  69 (6.8)  93 (9.1) |
| **Low physical activity, n (%)**^c^ | 4858 (19.5) | 765 (22.2) | 232 (22.8) |
| **Systolic blood pressure, mmHg** | 141.0 (20.0) | 140.0 (19.9) | 147.2 (19.9) |
| **Use of AHT, n (%)** | 4165 (16.7) | 440 (10.0) | 674 (60.1) |
| **BMI, kg/m^2^** | 25.7 (4.0) | 26.2 (4.3) | 27.0 (4.0) |
| **Incidence of events during follow up** |  |  |  |
| CAD, total events (events/1000 p-ys) | 2309 (6.7) | 483 (8.1) | 453 (39.8) |
| Ischemic Stroke, total events (events/1000 p-ys) | 1253 (3.6) | 296 (4.9) | 144 (11.4) |
| Cardiovascular mortality, events (events/1000 p-ys) | 1156 (3.2) | 295 (4.8) | 295 (21.5) |
| | Numbers are displayed as mean (SD) if not stated other  p-ys = person-years  ^a^ Subjects from MDCS excluded from the current study because of missing genetic and / or covariates data. Proportion numbers are based on available data in excluded subjects.  ^b^ One subject had missing data on prevalent CVD at baseline, and could therefore not be assigned a group.  ^c^ Defined as the lowest quintile of the Physical Activity score in MDCS (for definition of Physical Activity Score,  (see the methods section) | | --- | | | | |

**Table S2:** Main effects with adjustment for covariates

|  | CAD  HR (95 % CI) | Ischemic Stroke  HR (95 % CI) | Cardiovascular mortality  HR (95 % CI) |
| --- | --- | --- | --- |
| **rs4977574, per allele** | 1.16 (1.10-1.23) | 1.13 (1.04-1.22) | 1.16 (1.06-1.25) |
|  |  |  |  |
| **Smoking status**^a^ |  |  |  |
| Former smoker  Current smoker | 1.19 (1.07-1.32)  2.11 (1.90-2.35) | 1.01 (0.88-1.17)  1.73 (1.51-1.99) | 1.26 (1.08-1.46)  2.85 (2.45-3.30) |
|  |  |  |  |
| **Highest education**^a^  Elementary school (6-8 yrs)  Junior Sec. School (9-10 yrs)  Advanced level (12 yrs)  At least one additional year  University degree  *P for trend* | 0.99 (0.65-1.53)  0.87 (0.56-1.35)  0.84 (0.54-1.33)  0.86 (0.55-1.36)  0.75 (0.48-1.17)  *<0.001* | 0.53 (0.34-0.83)  0.48 (0.30-0.75)  0.42 (0.26-0.68)  0.45 (0.28-0.73)  0.42 (0.26-0.67)  *0.001* | 0.83 (0.47-1.47)  0.73 (0.41-1.30)  0.66 (0.36-1.21)  0.62 (0.33-1.14)  0.70 (0.37-1.22)  *0.002* |
| **Quintile of PA score**^a^  Q2  Q3  Q4  Q5 | 0.75 (0.66-0.85)  0.78 (0.69-0.89)  0.83 (0.73-0.94)  0.85 (0.75-0.96) | 0.76 (0.64-0.90)  0.77 (0.65-0.91)  0.70 (0.59-0.83)  0.73 (0.62-0.86) | 0.69 (0.58-0.83)  0.73 (0.61-0.87)  0.73 (0.62-0.88)  0.71 (0.60-0.84) |
| Models adjusted for main effect variables, age, sex, systolic blood pressure, body mass index and antihypertensive treatment.  PA score = Physical Activity Score  ^a^ Hazard ratio (HR) in relation to the first category in the categorical variables (never smokers, did not complete elementary school, and Q1 of PA-score respectively) | | | |

**Table S3:** Risk of incident CAD by rs4977574 stratified by smoking status

**Table S3A. Model adjusted for age (and sex):**

|  | Events (total cases included) | rs4977574 HR per allele | 95 % CI | P |
| --- | --- | --- | --- | --- |
| **Never smokers** | **675 (9642)** | **1.26** | **1.13-1.40** | **<0.001** |
| Men | 340 (2745) | 1.27 | 1.09-1.48 | 0.002 |
| Women | 335 (6895) | 1.24 | 1.07-1.45 | 0.005 |
|  |  |  |  |  |
| No passive smoking | 220 (3339) | 1.56 | 1.29-1.88 | <0.001 |
| Passive smoking | 379 (5069) | 1.14 | 0.99-1.32 | 0.068 |
|  |  |  |  |  |
| **Former smokers** | **814 (8300)** | **1.20** | **1.08-1.32** | **<0.001** |
| Men | 612 (4015) | 1.15 | 1.03-1.29 | 0.012 |
| Women | 202 (4285) | 1.31 | 1.08-1.59 | 0.006 |
|  |  |  |  |  |
| No passive smoking | 177 (2146) | 1.30 | 1.05-1.60 | 0.015 |
| Passive Smoking | 528 (5221) | 1.19 | 1.06-1.35 | 0.004 |
|  |  |  |  |  |
| **Current smokers** | **820 (7000)** | **1.05** | **0.95-1.16** | **0.326** |
| Men | 520 (2693) | 1.06 | 0.94-1.20 | 0.329 |
| Women | 300 (4307) | 1.03 | 0.87-1.21 | 0.743 |

**Table S3B. Model adjusted for age, (sex), SBP, BMI, AHT, Education, Quintiles of PA:**

|  | Events (total cases included) | rs4977574 HR per allele | 95 % CI | P |
| --- | --- | --- | --- | --- |
| **Never smokers** | **675 (9642)** | **1.24** | **1.11-1.38** | **<0.001** |
| Men | 340 (2745) | 1.26 | 1.08-1.47 | 0.004 |
| Women | 335 (6895) | 1.23 | 1.06-1.43 | 0.006 |
|  |  |  |  |  |
| No passive smoking | 220 (3339) | 1.53 | 1.26-1.85 | <0.001 |
| Passive smoking | 379 (5069) | 1.12 | 0.97-1.29 | 0.116 |
|  |  |  |  |  |
| **Former smokers** | **814 (8300)** | **1.21** | **1.10-1.34** | **<0.001** |
| Men | 612 (4015) | 1.18 | 1.05-1.32 | 0.004 |
| Women | 202 (4285) | 1.33 | 1.09-1.61 | 0.004 |
|  |  |  |  |  |
| No passive smoking | 177 (2146) | 1.31 | 1.06-1.61 | 0.012 |
| Passive smoking | 528 (5221) | 1.22 | 1.08-1.38 | 0.001 |
|  |  |  |  |  |
| **Current smokers** | **820 (7000)** | **1.05** | **0.95-1.15** | **0.368** |
| Men | 520 (2693) | 1.06 | 0.94-1.20 | 0.362 |
| Women | 300 (4307) | 1.03 | 0.88-1.21 | 0.729 |

**Table S4:** CVD Mortality by rs4977574 stratified by smoking status

**Table S4A. Model adjusted for age (and sex):**

|  | Events (total cases included) | rs4977574 HR per allele | 95 % CI | P |
| --- | --- | --- | --- | --- |
| **Never smokers** | **327 (9642)** | **1.40** | **1.20-1.63** | **<0.001** |
| Men | 137 (2745) | 1.35 | 1.07-1.72 | 0.013 |
| Women | 190 (6895) | 1.43 | 1.17-1.75 | <0.001 |
|  |  |  |  |  |
| No passive smoking | 113 (3339) | 1.78 | 1.37-2.32 | <0.001 |
| Passive smoking | 174 (5070) | 1.27 | 1.03-1.57 | 0.025 |
|  |  |  |  |  |
| **Former smokers** | **383 (8297)** | **1.05** | **0.91-1.21** | **0.525** |
| Men | 270 (4011) | 0.98 | 0.83-1.16 | 0.807 |
| Women | 113 (4284) | 1.22 | 0.94-1.58 | 0.136 |
|  |  |  |  |  |
| No passive smoking | 88 (2145) | 1.38 | 1.02-1.85 | 0.034 |
| Passive smoking | 247 (5218) | 0.96 | 0.81-1.15 | 0.676 |
|  |  |  |  |  |
| **Current smokers** | **446 (7000)** | **1.08** | **0.94-1.23** | **0.270** |
| Men | 265 (2689) | 1.08 | 0.91-1.28 | 0.362 |
| Women | 181 (4307) | 1.07 | 0.86-1.32 | 0.552 |

**Table S4B. Model adjusted for age, (sex), SBP, BMI, AHT, Education, Quintiles of PA:**

|  | Events (total cases included) | rs4977574 HR per allele | 95 % CI | P |
| --- | --- | --- | --- | --- |
| **Never smokers** | **327 (9642)** | **1.38** | **1.18-1.61** | **<0.001** |
| Men | 137 (2745) | 1.33 | 1.04-1.70 | 0.021 |
| Women | 190 (6895) | 1.44 | 1.18-1.76 | <0.001 |
|  |  |  |  |  |
| No passive smoking | 113 (3339) | 1.71 | 1.31-2.24 | <0.001 |
| Passive smoking | 174 (5070) | 1.28 | 1.04-1.57 | 0.023 |
|  |  |  |  |  |
| **Former smokers** | **383 (8297)** | **1.05** | **0.91-1.21** | **0.489** |
| Men | 270 (4011) | 0.99 | 0.83-1.17 | 0.870 |
| Women | 113 (4284) | 1.22 | 0.94-1.58 | 0.128 |
|  |  |  |  |  |
| No passive smoking | 88 (2145) | 1.36 | 1.01-1.84 | 0.042 |
| Passive smoking | 247 (5218) | 0.98 | 0.82-1.17 | 0.824 |
|  |  |  |  |  |
| **Current smokers** | **446 (7000)** | **1.09** | **0.95-1.24** | **0.212** |
| Men | 265 (2689) | 1.09 | 0.92-1.30 | 0.312 |
| Women | 181 (4307) | 1.08 | 0.87-1.33 | 0.498 |

**Table S5:** Interactions between rs4977574 and smoking, education and physical activity on end points

|  | CAD | Ischemic Stroke | Cardiovascular  mortality |
| --- | --- | --- | --- |
|  | **P _interaction_** | **P _interaction_** | **P _interaction_** |
| **Crude model** ^a^ |  |  |  |
| Smoking status | **0.035** | 0.702 | **0.012** |
| Highest Education | 0.082 | 0.876 | 0.681 |
| Physcial activty | 0.457 | 0.251 | 0.286 |
|  |  |  |  |
| **Adjusted model** ^b^ |  |  |  |
| Smoking status | **0.035** | 0.569 | **0.029** |
| Highest Education | 0.080 | 0.924 | 0.696 |
| Physcial activty | 0.565 | 0.217 | 0.372 |

P-values based on Chi 2 distribution from likelihood ratio tests comparing model fit with and without the interaction terms.

^a^ Model adjusted for age and sex

^b^ Model adjusted for covariates age, sex, systolic blood pressure, body mass index, antihypertensive treatment and (smoking), (education), (quintiles of physical activity)

**Table S6:** Smoking as a risk factor for incident CAD, stratified by number of rs4977574 risk alleles

**Table S6A. Model adjusted for age (and sex):**

| rs4977574 risk alleles | Events (total cases included) | Former smoker HR (95 % CI) | P | Current smoker HR (95% CI) | P |
| --- | --- | --- | --- | --- | --- |
| **0** | **633 (7608)** | **1.24 (1.01-1.52)** | **0.038** | **2.21 (1.81-2.70)** | **<0.001** |
| Men | 412 (2881) | 1.41 (1.09-1.83) | 0.010 | 2.29 (1.75-2.99) | <0.001 |
| Women | 221 (4727) | 1.02 (0.72-1.45) | 0.908 | 2.25 (1.66-3.05) | <0.001 |
|  |  |  |  |  |  |
| **1** | **1134 (12309)** | **1.24 (1.07-1.45)** | **0.005** | **2.21 (1.90-2.56)** | **<0.001** |
| Men | 717 (4680) | 1.18 (0.98-1.43) | 0.087 | 1.97 (1.62-2.39) | <0.001 |
| Women | 417 (7629) | 1.40 (1.09-1.80) | 0.009 | 2.66 (2.12-3.33) | <0.001 |
|  |  |  |  |  |  |
| **2** | **542 (5024)** | **1.12 (0.92-1.38)** | **0.265** | **1.48 (1.19-1.84)** | **<0.001** |
| Men | 343 (1892) | 1.18 (0.91-1.54) | 0.215 | 1.57 (1.18-2.09) | 0.002 |
| Women | 199 (3132) | 1.13 (0.80-1.58) | 0.496 | 1.43 (1.01-2.02) | 0.043 |

**TableS6B. Model adjusted for age, (sex), SBP, BMI, AHT, Education, Quintiles of PA:**

| rs4977574 risk alleles | Events (total cases included) | Former smoker HR (95 % CI) | P | Current smoker HR (95% CI) | P |
| --- | --- | --- | --- | --- | --- |
| **0** | **633 (7608)** | **1.17 (0.95-1.43)** | **0.141** | **2.29 (1.87-2.80)** | **<0.001** |
| Men | 412 (2881) | 1.29 (0.99-1.68) | 0.059 | 2.32 (1.77-3.04) | <0.001 |
| Women | 221 (4727) | 1.05 (0.74-1.50) | 0.783 | 2.38 (1.75-3.25) | <0.001 |
|  |  |  |  |  |  |
| **1** | **1134 (12309)** | **1.23 (1.06-1.43)** | **0.007** | **2.34 (2.01-2.71)** | **<0.001** |
| Men | 717 (4680) | 1.14 (0.95-1.38) | 0.170 | 2.02 (1.66-2.45) | <0.001 |
| Women | 417 (7629) | 1.44 (1.12-1.85) | 0.005 | 2.99 (2.38-3.76) | <0.001 |
|  |  |  |  |  |  |
| **2** | **542 (5024)** | **1.12 (0.91-1.37)** | **0.301** | **1.55 (1.24-1.93)** | **<0.001** |
| Men | 343 (1892) | 1.14 (0.87-1.49) | 0.338 | 1.58 (1.18-2.11) | 0.002 |
| Women | 199 (3132) | 1.18 (0.84-1.66) | 0.339 | 1.55 (1.09-2.20) | 0.016 |

**Table S7:** Smoking as a risk factor for CVD Mortality, stratified by number of rs4977574 risk alleles

**TableS7A. Model adjusted for age (and sex):**

| rs4977574 risk alleles | Events (total cases included) | Former smoker HR (95 % CI) | P | Current smoker HR (95% CI) | P |
| --- | --- | --- | --- | --- | --- |
| **0** | **310 (7604)** | **1.76 (1.30-2.38)** | **<0.001** | **3.35 (2.48-4.52)** | **<0.001** |
| Men | 190 (2877) | 1.97 (1.30-2.99) | 0.002 | 3.32 (2.15-5.12) | <0.001 |
| Women | 120 (4726) | 1.49 (0.93-2.40) | 0.099 | 3.62 (2.38-5.50) | <0.001 |
|  |  |  |  |  |  |
| **1** | **586 (12309)** | **1.20 (0.96-1.48)** | **0.106** | **2.77 (2.26-3.39)** | **<0.001** |
| Men | 343 (4676) | 1.14 (0.85-1.52) | 0.380 | 2.69 (2.03-3.57) | <0.001 |
| Women | 243 (7629) | 1.33 (0.95-1.85) | 0.098 | 2.85 (2.13-3.81) | <0.001 |
|  |  |  |  |  |  |
| **2** | **260 (5024)** | **1.04 (0.77-1.42)** | **0.781** | **2.00 (1.48-2.70)** | **<0.001** |
| Men | 139 (1892) | 1.06 (0.69-1.62) | 0.803 | 2.03 (1.31-3.14) | 0.002 |
| Women | 121 (3130) | 1.09 (0.70-1.71) | 0.708 | 2.02 (1.32-3.10) | 0.001 |

**Table S7B. Model adjusted for age, (sex), SBP, BMI, AHT, Education, Quintiles of PA:**

| rs4977574 risk alleles | Events (total cases included) | Former smoker HR (95 % CI) | P | Current smoker HR (95% CI) | P |
| --- | --- | --- | --- | --- | --- |
| **0** | **310 (7604)** | **1.68 (1.24-2.29)** | **0.001** | **3.43 (2.53-4.65)** | **<0.001** |
| Men | 190 (2877) | 1.83 (1.20-2.80) | 0.005 | 3.29 (2.12-5.11) | <0.001 |
| Women | 120 (4726) | 1.54 (0.95-2.48) | 0.080 | 3.72 (2.43-5.71) | <0.001 |
|  |  |  |  |  |  |
| **1** | **586 (12309)** | **1.20 (0.96-1.49)** | **0.108** | **2.95 (2.40-3.63)** | **<0.001** |
| Men | 343 (4676) | 1.08 (0.81-1.45) | 0.583 | 2.70 (2.03-3.59) | <0.001 |
| Women | 243 (7629) | 1.37 (0.98-1.92) | 0.064 | 3.21 (2.38-4.33) | <0.001 |
|  |  |  |  |  |  |
| **2** | **260 (5024)** | **1.05 (0.78-1.43)** | **0.738** | **2.15 (1.58-2.94)** | **<0.001** |
| Men | 139 (1892) | 1.00 (0.65-1.54) | 0.985 | 2.12 (1.36-3.32) | 0.001 |
| Women | 121 (3130) | 1.17 (0.74-1.84) | 0.502 | 2.21 (1.42-3.43) | <0.001 |
